# Mubritinib enhanced the inhibiting function of cisplatin in lung cancer by interfering with mitochondrial function

**DOI:** 10.1111/1759-7714.14425

**Published:** 2022-04-16

**Authors:** Jingyao Dong, Dongshan Zhu, Mengmeng Chen, Taiwei Wang, Yan Gao, Wei Liu

**Affiliations:** ^1^ Department of Thoracic Surgery The First Hospital of Jilin University Changchun China

**Keywords:** lung cancer, mitochondrial electron transport chain, mubritinib, PI3K/mTOR pathway, ROS

## Abstract

**Background:**

Lung cancer is one of the most lethal cancers worldwide. Cisplatin, a widely used anti‐lung cancer drug, has been limited in clinical application due to its drug resistance. Medicines targeting mitochondrial electron transport chain (ETC) complexes may be effective candidates for cisplatin‐based chemotherapy.

**Methods:**

In this study, the small molecule drug library from Food and Drug Administration FDA was used to screen for medicines targeting ETC. MTT and colony formation assays were used to investigate cell proliferation. Flow cytometry was employed to analyze cell cycle, apoptosis, reactive oxygen species (ROS), and mitochondrial membrane potential. Wound scratch and transwell assays were used to detect migration and invasion abilities. The activities of the ETC complex were tested using kits. Western blot analysis was used to investigate the expressions of related proteins. A mouse xenograft model was constructed to verify the antitumor effect in vivo.

**Results:**

The results showed that mubritinib can reduce the activation of the PI3K/mTOR signal pathway, disrupt mitochondrial function, significantly increase ROS levels and induce oxidative stress, and ultimately exert its antitumor effect against non‐small cell lung cancer (NSCLC) both in vivo and in vitro. In addition, the combination of cisplatin and mubritinib can improve the tumor‐suppressive effect of cisplatin.

**Conclusion:**

Mubritinib can upregulate intracellular ROS concentration and cell apoptosis, inhibit the PI3K signaling pathway and interfere with the function of mitochondria, thus reducing cell proliferation and increasing ROS induced apoptosis by reducing the activation of Nrf2 by PI3K.

## Graphical abstract

In addition to directly causing DNA damage, cisplatin can also lead to DNA damage by inducing ROS production, thus leading to apoptosis. However, ROS can activate Nrf2, which in turn leads to the increase of HO‐1, GPX4 and other antioxidants, which feedback the inhibitory effect of ROS. In addition, the highly expressed PI3K/Akt/mTOR signaling pathway in tumor cells promotes energy generation and cell proliferation by promoting mitochondrial function. PI3K also inhibited Nrf2 activation. This might be one of the important mechanisms of endogenous drug resistance of cisplatin. Mubritinib can upregulate intracellular ROS concentration and cell apoptosis on the one hand, while on the other hand, it can inhibit the PI3K signaling pathway and interfere with the function of mitochondria, thus reducing the cell proliferation and increasing ROS induced apoptosis by reducing the activation of Nrf2 by PI3K.

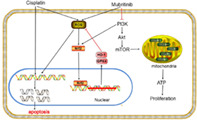

## INTRODUCTION

Lung cancer (LC) is the second most common cancer and one of the most lethal cancers worldwide.^1^ Non‐small cell lung cancer (NSCLC) is the most common type of LC and accounts for approximately 85% of cases of LC. Due to the poor specificity of symptoms in NSCLC, 75% of patients are in advanced stages at the time of diagnosis^2^ with a 1‐year survival ratio of only 10%–15%.^2^

Cisplatin, a first‐line medicine for the treatment of NSCLC, is thought to block cell cycle progression and induce cell death by binding to nuclear DNA and inducing cisplatin‐DNA cross‐linking.^3^ However, the clinical efficacy of cisplatin is severely limited by drug resistance, which is mainly related to the increased DNA self‐repair.^3^, ^4^ Recent studies have found that LC cell death can be induced by directly interacting with mitochondria.^5^ Mitochondria are considered as a key subcellular compartment that regulates cellular apoptosis by producing excess ROS.^6^ Mitochondria‐associated ROS overproduction is mainly due to the dysfunction of mitochondrial respiration, which is associated with the inhibition of mitochondrial electron transport chain (ETC) complexes‐I and III.^6^ Therefore, medicines targeting mitochondrial ETC complexes may be effective candidates for cisplatin‐based chemotherapy.

A recent study found that mubritinib, a Food and Drug Administration (FDA) approved drug initially used as a human epidermal growth factor receptor 2 (HER2) inhibitor, could significantly reduce the development of leukemia through inducing mitochondrial respiration‐associated ROS overproduction and cell apoptosis instead of targeting HER2 protein.^7^ Additionally, further studies demonstrated that mubritinib acts on mitochondrial complex I to inhibit the production of ATP.^7^ However, the effect of mubritinib on solid tumors remains unknown. In this study, the antitumor effects of mubritinib in NSCLC and its influence on mitochondrial ETC were investigated. In addition, the sensitization effect of mubritinib on the antitumor effect of cisplatin was observed to provide evidence for future application of mubritinib in cisplatin‐based chemotherapy.

## METHODS

### Cell culture

Human lung adenocarcinoma cell lines NCI‐H1975, A549, HCC‐827, and NCI‐H2170 were purchased from ATCC and cultured in a Dulbecco's Modified Eagle's Medium (HyClone) containing 10% fetal bovine serum and 1‰ tertiary antibody in a CO_2_ incubator at 37°C.

### MTT and plate colony formation assay

The small‐molecule drug library from FDA was used to investigate the sensitivity of NCI‐H1975 using the MTT assay. The antitumor effects of mubritinib on NCI‐H1975, A549, HCC‐827, and NCI‐H2170 cells were observed using MTT assay after different doses of mubritinib treatment for 24, 48, and 72 h. NCI‐H1975 cells seeded in 6‐well plates were treated with mubritinib and/or cisplatin for 15 days and stained with crystal violet solution.

### Flow cytometry

After 48 h of treatment with mubritinib and/or cisplatin, NCI‐H1975 cells were harvested and washed with PBS. Some cells were stained with Annexin V‐FITC and PI for 15 min in the dark at room temperature (RT) and then analyzed for apoptosis using FACScan (Becton Dickinson). Some cells were fixed with 75% cold ethanol at −20°C for 1 h, stained with 200 μl PI (50 μg/ml) for 30 min at RT in the dark, and the cell cycles were analyzed using FACScan. Some cells were stained with 500 μl JC‐1 (20 μg/ml) for 20 min at 37°C and analyzed for the mitochondrial membrane potential using FACScan. Other cells were stained with 500 μl of DCHF‐DA working solution for 30 min at 37°C and analyzed for ROS using FACScan.

### Scratch‐wound assay and transwell invasion assay

NCI‐H1975 cells seeded in 12‐well plates were scratched using a sterile 0.4–0.5 mm wide pipette tip. After 48 h treatment with mubritinib and/or cisplatin, the wounds were observed at 0 and 48 h after scratching.

For NCI‐H1975 cells, the serum‐free cell suspension was added to the upper chamber and the serum‐containing medium was added to the lower chamber. After being treated with mubritinib and/or cisplatin for 48 h, the lower surface was fixed with 70% methanol solution for 30 min, stained with crystal violet, and examined under the microscope.

### RNA sequencing

NCI‐H1975 cells treated with 80 nM mubritinib for 24 h were harvested and subjected to RNA sequencing (RNA‐seq) performed by Huada Gene Corporation.

### Determination of mitochondrial respiratory chain complex activity

After being treated with mubritinib and/or cisplatin for 48 h, NCI‐H1975 cells were harvested and resuspended with mitochondrial isolation reagent. Cellular mitochondria were extracted and the activities of mitochondrial respiratory chain complex I‐V were detected according to the manufacturer's instruction.

### Western blot assay

After being treated with mubritinib and/or cisplatin for 48 h, NCI‐H1975 cells were collected and lysed in RIPA lysis buffer containing phosphatase and protease inhibitors. After BCA quantification, proteins were separated by 12% SDS‐PAGE and transferred to methanol‐activated PVDF membranes. Membranes were blocked with 5% skimmed milk for 1 h, followed by incubation with primary antibodies (anti‐ PI3K, mTOR, Akt, p‐Akt, ERK, p‐ERK, NRF‐2, HO‐1, GPX4, and tublin) (Affinity company) overnight at 4°C, with HRP‐conjugated secondary antibodies for 1 h at RT, visualized using ECL, and quantified with Image J Software.

### Mouse subcutaneous transplanted tumor model and drug administration

6‐week‐old male BALB/c‐Nu mice were purchased from Beijing Unilever Laboratory Animal Co., Ltd., and raised under specific nonpathogenic conditions. The feeding and operation of experimental animals followed regulations on the management of experimental animals of Jilin University, and the experimental protocol was approved by the Experimental Animal Ethics Committee of Jilin University. Animal care was carried out following the recommendations in the ARRIVE guidelines.NCI‐H1975 cells 2 × 10^6^ were subcutaneously injected into the right flank of each mouse, and the growth of the transplanted tumor was continuously monitored. When the tumor volume reached 100–150 mm^3^, the mice were divided into four groups with five mice in each group: (1) control (Ctr): intraperitoneal injection of 100 μl of PBS once a day; (2) cisplatin (Pt): intraperitoneal injection of 100 μl of cisplatin (5 mg/kg), once a week; (3) mubritinib (Mu): intraperitoneal injection of 100 μl of mubritinib (10 mg/kg), once a day; and (4) combination (Com): combined injection of cisplatin and mubritinib as the monotherapy group.

During treatment, the long and short diameters of the transplanted tumor were measured, and the volumes were calculated: volume = short diameter^2^ × long diameter/2. At the end of the treatment, mice were sacrificed by inhalation of an overdose of ether. The tumor, heart, liver, spleen, lung, and kidney were dissected and fixed with formalin.

### Morphological detection

Fixed tissues were dehydrated and embedded. The resulting paraffin blocks were prepared and sliced into 4 μm slices. After being dewaxed and hydrated, these slices were stained with hematoxylin and eosin. Later, the slices were destained in ethanol and viewed under a microscope.

For immunohistochemical staining, hydrated slices were heated in citrate antigen, incubated with drops of peroxidase blocker, sealed with goat serum, and incubated in primary antibody overnight and biotin‐labeled secondary antibody for 20 min. After incubation in antibiotic peroxidase solution and DAB solution for the color development, nuclei were stained with hematoxylin.

For TUNEL staining, hydrated slices were incubated with DNase‐free protease K and TUNEL solution, and visualized under the immunofluorescence microscope. Five fields were randomly selected and the number of cells was counted. Apoptosis rate = the number of apoptotic cells/the total number of tumor cells ×100%.

### Statistical analysis

All data are expressed as mean ± standard deviation (mean ± SEM). One‐way ANOVA in SPSS software was used for comparison between multiple groups, and *p* < 0.05 was considered statistically significant.

## RESULTS

### Screening of small molecule drugs that inhibit LC cells

**FIGURE 1:**
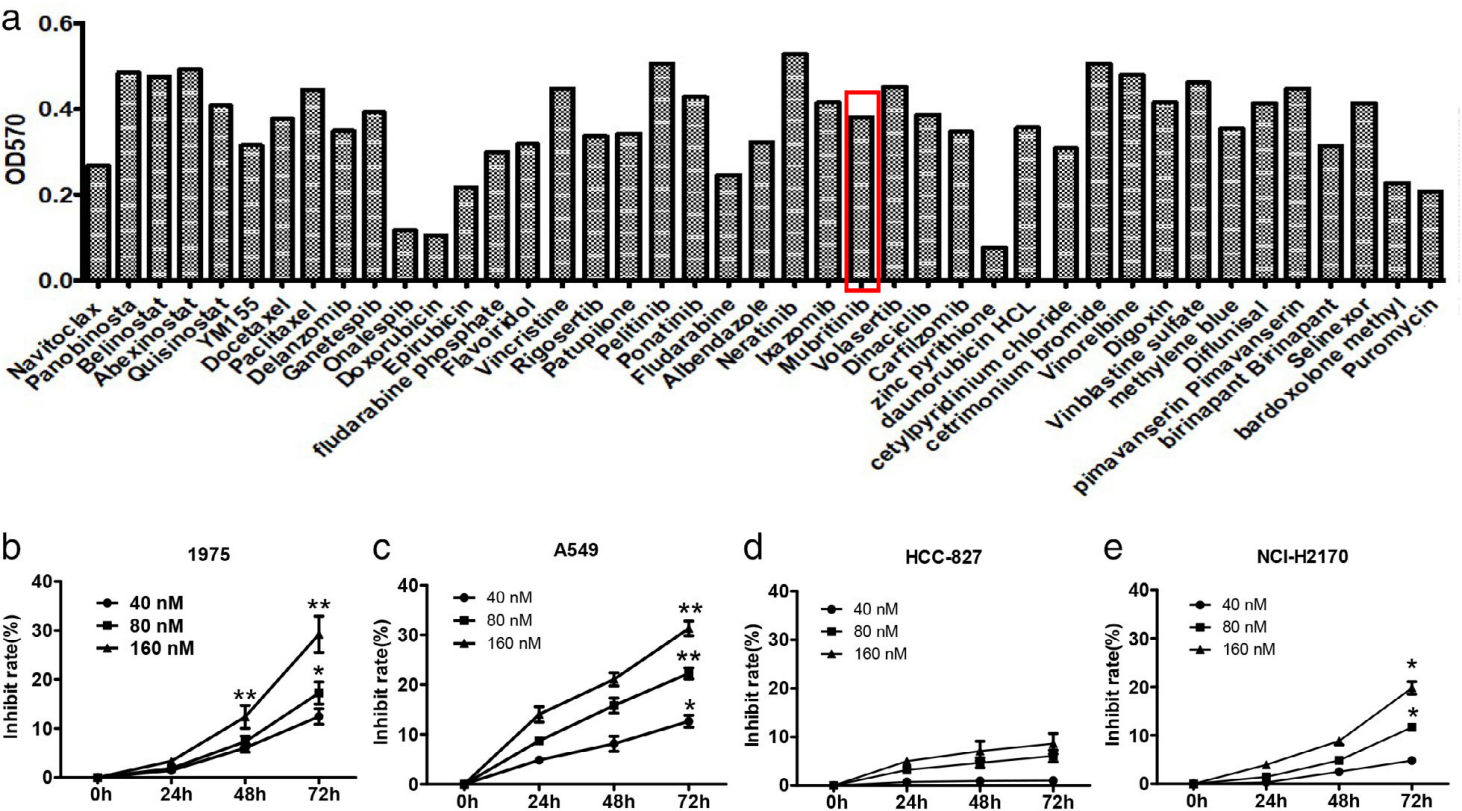
Screening of small molecule drugs that inhibit lung cancer cells. (a) Screening of small molecule drugs that inhibit lung neoplasms using FDA drug library. (b–e) Inhibitory effect of mubritinib on four lung cancer cell lines NCI‐H1975, A549, HCC‐827, and NCI‐H2170

The FDA drug library search results showed that 42 out of 2992 small molecules had significant inhibitory effects on NCI‐H1975 lung neoplasms cells (Figure 1a). Mubritinib was selected for this study since it showed similar antitumor activity to docetaxel but no research report on lung neoplasms inhibition so far.

Next, the inhibitory effects of mubritinib on NCIH1975, A549, HCC‐827, and NCI‐H2170 were detected by MTT. The results showed that the antitumor effects of mubritinib on NCI‐H1975 and A549 were better than that of HCC‐827 and NCI‐H2170 (Figure 1b–e). In addition, the inhibition rates of the four cells were increased with increasing drug concentration and time (Figure 1b–e).

### Inhibitory effect of mubritinib on LC cells

**FIGURE 2:**
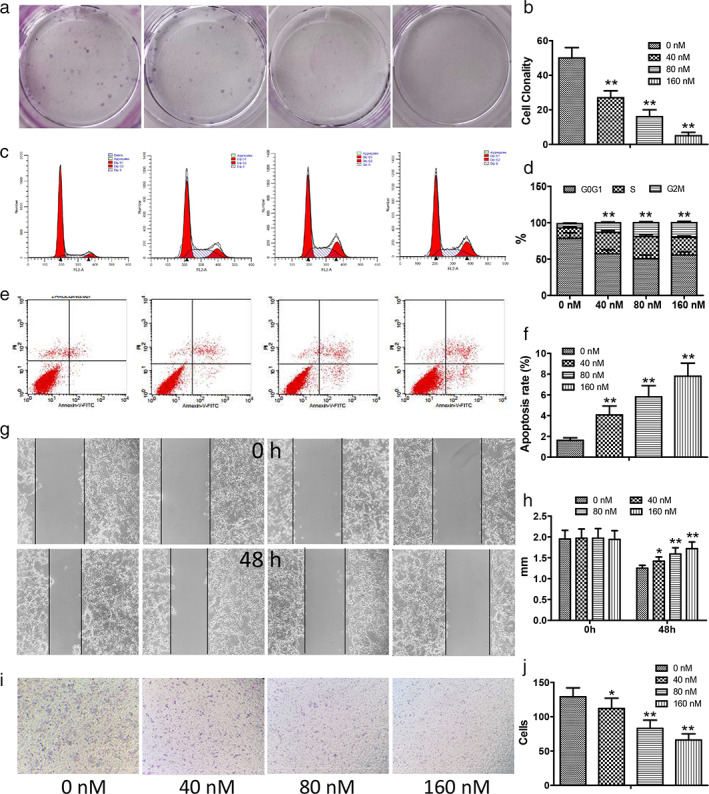
Inhibitory effect of mubritinib on NCI‐H1975 cells. (a,b) Proliferation of NCI‐H1975 cells treated with mubritinib as detected by plate colony formation assay. (c,d) The cell cycle proportion of NCI‐H1975 cells by FACScan. (e,f) NCI‐H1975 cells apoptosis using FACScan. (g,h) The migration ability of NCI‐H1975 cells when treated with different concentrations of mubritinib using the scratch assay. (i,j) The invasion ability of NCI‐H1975 cells using transwell assay

The results of the plate colony formation assay showed that the proliferation of NCI‐H1975 decreased in a dose‐dependent manner (*p* < 0.01) (Figure 2a,b). In addition, cell cycle assay indicated that the proportion of NCI‐H1975 cells in the G2 phase was significantly increased after mubritinib treatment (*p* < 0.01) (Figure 2c,d).

The results of Annexin V/PI staining flow cytometry showed that mubritinib could induce apoptosis of NCI‐H1975 cells with a significant dose‐effect relationship (*p* < 0.01) (Figure 2e,f). After being treated with mubritinib for 48 h, the migration and invasion abilities of cells in mubritinib‐treated groups were significantly reduced (*p* < 0.05, *p* < 0.01) (Figure 2g–j) as shown in the scratch‐wound and transwell assays.

### Potential mechanism of action of mubritinib in LC cells

**FIGURE 3:**
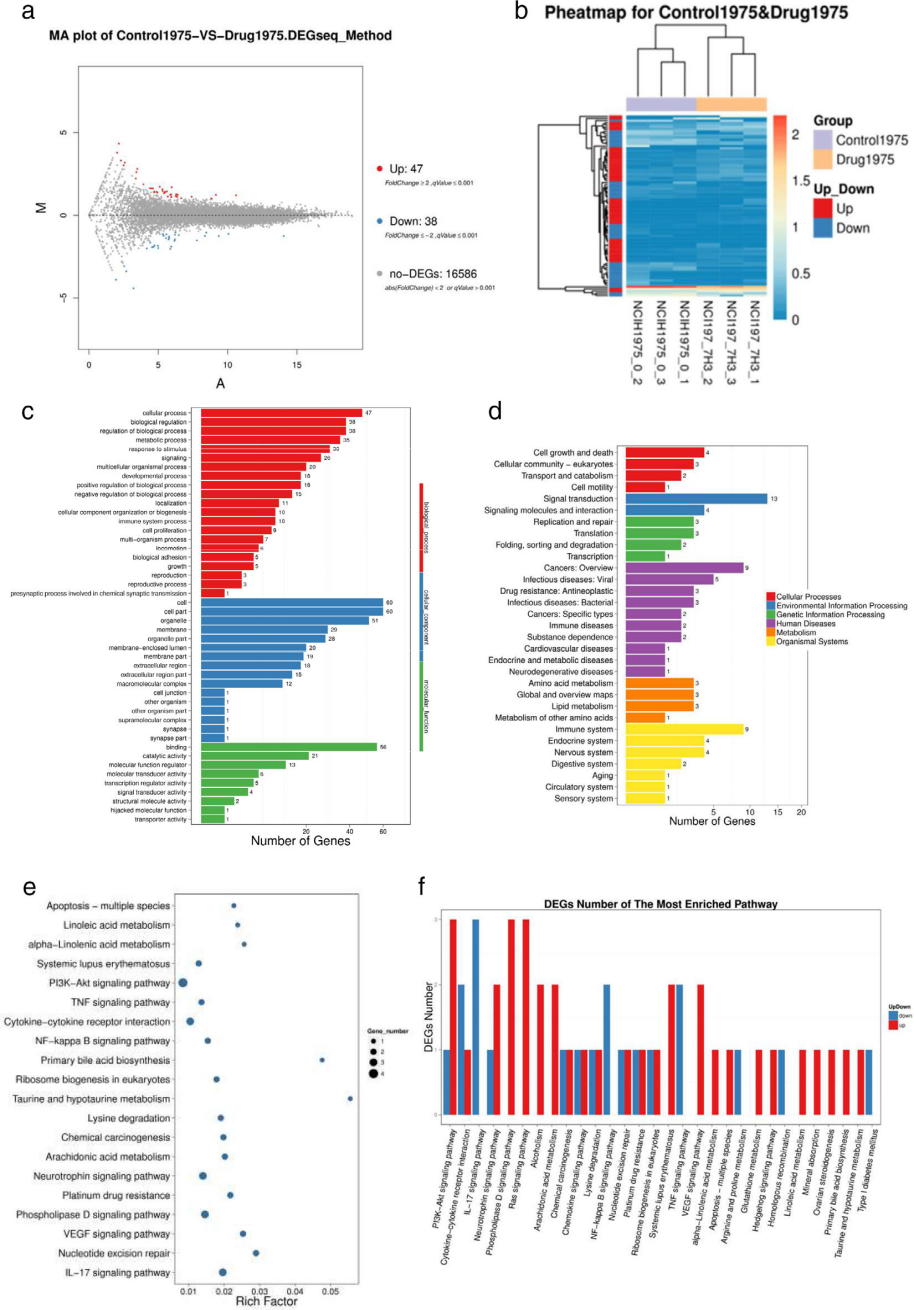
The potential mechanism of action of mubritinib on lung cancer cells. (a,b) The up‐ and downregulated genes as shown using RNA‐seq analysis. (c) GO enrichment analysis indicated the most enriched pathways related to the significantly changed genes. (d) KEGG enrichment analysis showed the cellular and biological regulation processes associated with the significantly changed genes. (e) The significantly changed biological activities. (f) Changes in signaling pathways activated after mubritinib treatment

RNA‐seq results showed that 47 and 38 genes were significantly up‐ and downregulated (fold change >2, *q* vaule ≤0.001) in NCI‐H1975 cells treated with mubritinib, respectively (Figure 3a,b and Appendix S1). GO enrichment analysis showed that TXNIP was the most variable gene (Figure 3c), and KEGG enrichment analysis showed differences in the cellular and biological regulation processes, such as metabolism, proliferation, immunity, and adhesion (Figure 3d). Six biological activities, including cellular process, environmental information processing, genetic information processing, human diseases, metabolism, and organic systems, were significantly changed in mubritinib treated cells (Figure 3e). The activation of signaling pathways such as PI3K/Akt, IL‐17, phospholipase D, Ras, and NF‐κB was altered after treatment with mubritinib (Figure 3f).

### Effects of mubritinib on mitochondrial function

**FIGURE 4:**
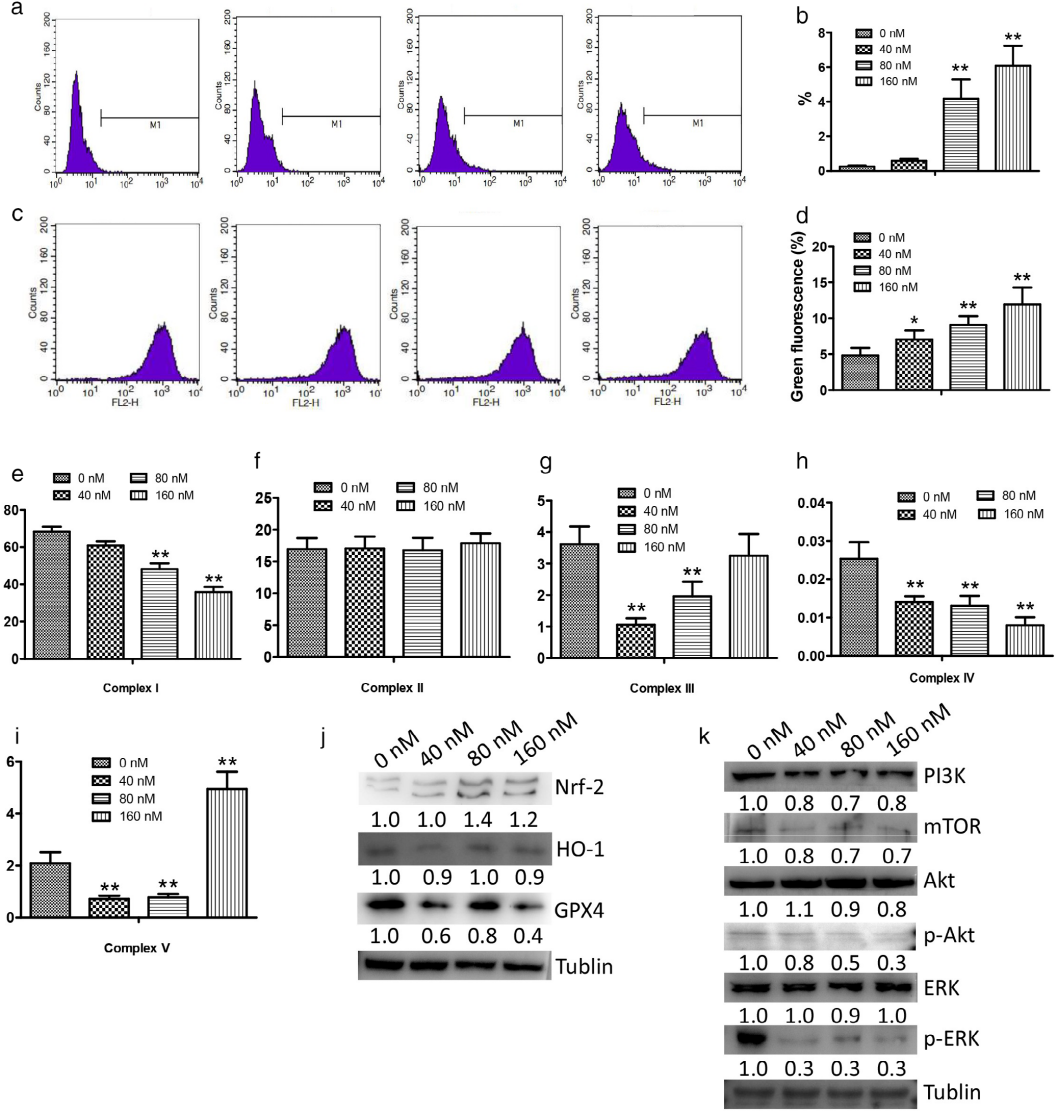
Effects of mubritinib on mitochondrial function in NCI‐H1975 cells. (a,b) ROS as detected by flow cytometry. (c,d) JC‐1 as detected using flow cytometry. (e–i) Activation of mitochondrial respiratory chain complex enzyme I, II, III, IV, and V. (j) Expressions of Nrf‐2, HO‐1, and GPX4 as detected using Western blot. (k) Expressions of PI3K, mTOR, Akt, p‐Akt, ERK, and p‐ERK as detected using Western blot

To investigate the molecular mechanism of mubritinib on NCI‐H1975 cells, we detected the expressions of PI3K, mTOR, Akt, and ERK proteins. As shown in Figure 4k, the expressions of PI3K, mTOR, p‐Akt, and p‐ERK were significantly decreased after treatment with mubritinib, suggesting that mubritinib may block the activation of the PI3K‐Akt–mTOR pathway in NCI‐H1975 cells.

mTOR is an important protein involved in the regulation of cellular energy metabolism and macromolecule synthesis. In this study, the mitochondrial respiratory chain complex enzyme I, III, IV, and V involved in ATP energy synthesis showed a significant decrease after mubritinib treatment (*p* < 0.05), suggesting that mubritinib can cause mitochondrial dysfunction and lead to abnormal energy metabolism (Figure 4e–i).

The abnormal activity of mitochondrial complex enzymes affects electron transport in the respiratory chain and induces the production of ROS. As shown in Figure 4a–d, ROS increased and more JC‐1 fluorescent turned green with the increasing dose of mubritinib (*p* < 0.01), indicating that mubritinib increased the production of ROS, which in turn promoted the reduction of mitochondrial membrane potential.

The expression levels of Nrf‐2, HO‐1, and GPX4 were later detected. The results showed that the expression of Nrf‐2 was significantly upregulated after treatment with mubritinib, whereas the HO‐1 and GPX4 were downregulated with increasing dose (*p* < 0.05) (Figure 4j), suggesting that the ROS induced by mubritinib could activate antioxidant pathways and deplete antioxidant‐related proteins.

### Mubritinib promotes the inhibitory effect of cisplatin

**FIGURE 5:**
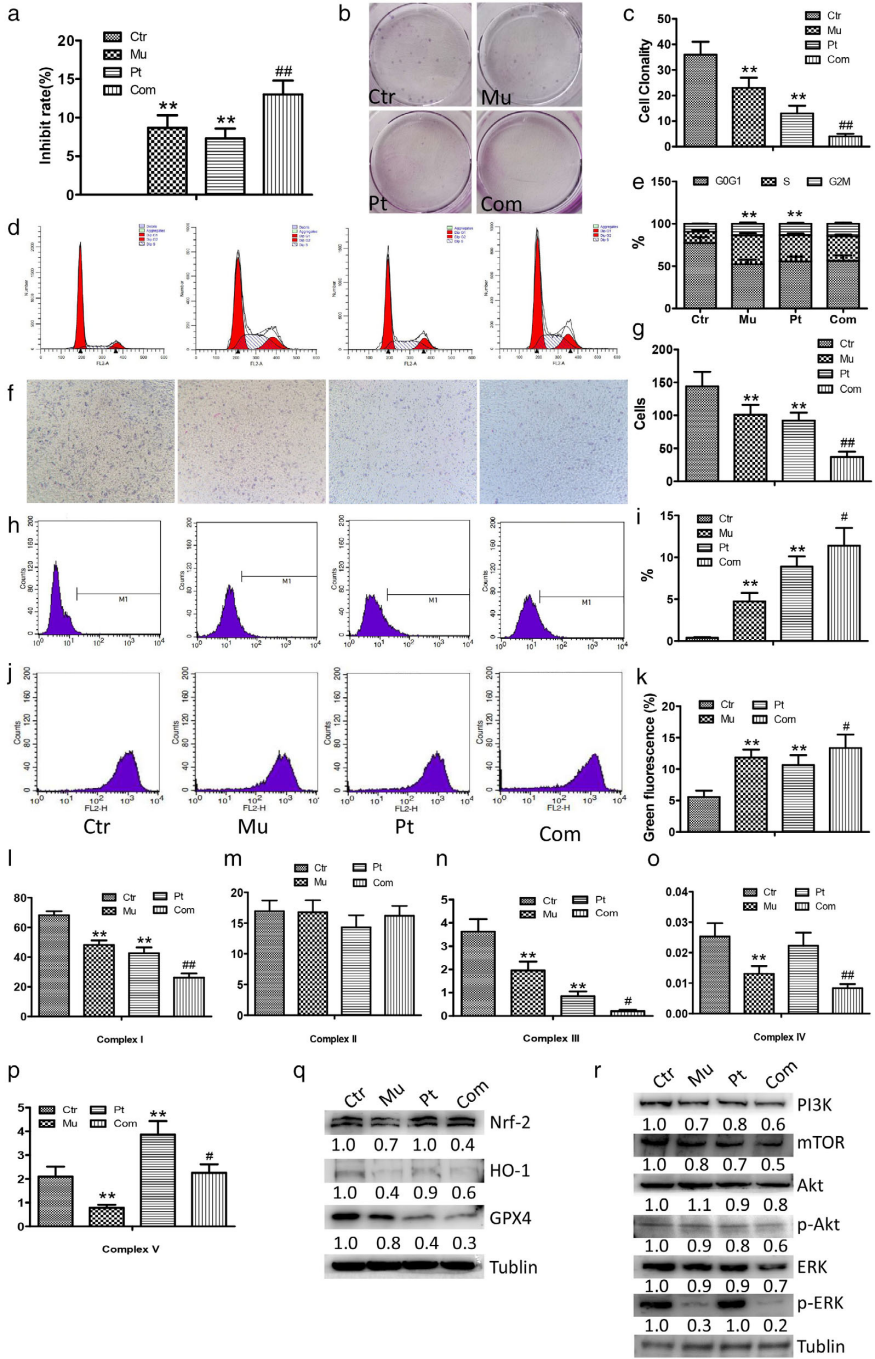
Mubritinib promoted the inhibitory effect of cisplatin in NCI‐H1975 cells. (a) The inhibition rate of mubritinib combined with cisplatin as detected using MTT assay. (b,c) The proliferation of NCI‐H1975 cells was detected using plate clone assay. (d,e) The cell cycle proportion of NCI‐H1975 cells using FACScan. (f, g) The invasion ability of NCIH1975 cells using transwell assay. (h,i) ROS as detected using flow cytometry. (j, k) JC‐1 as detected using flow cytometry. (l–p) Activation of mitochondrial respiratory chain complex enzyme I, II, III, IV, and V. (q) Nrf‐2, HO‐1, and GPX4 expression as detected using Western blot. (r) PI3K, mTOR, Akt, p‐Akt, ERK, and pERK expression as detected using Western blot

To clarify whether mubritinib can increase the inhibitory effect of cisplatin on NCI‐H1975 cells, we performed MTT and clonal formation assays. The results showed that the inhibition rate of 80 nM mubritinib combined with 0.1 μM cisplatin was significantly higher than that of the single‐drug group (*p* < 0.05) (Figure 5a–c). In addition, cell cycle results showed that both the mubritinib group and the cisplatin group had G2 phase arrest (*p* < 0.01), but there was no significant difference between the combination group and the single‐dose administration group (*p* > 0.05) (Figure 5d,e). In addition, the invasion ability decreased in the monotherapy groups (*p* < 0.05), and was further reduced in the combined group (*p* < 0.01) (Figure 5f,g).

The results of ROS detection showed that ROS levels of NCI‐H1975 cells increased in both mubritinib and cisplatin groups (*p* < 0.01), and further increased in the combined group (*p* < 0.05) (Figure 5h,i). The JC‐1 detection showed that the mitochondrial membrane potential significantly decreased in the monotherapy group (*p* < 0.05) and further decreased in the combined group (*p* < 0.01) (Figure 5j,k).

In addition, the activity of complexes I, III, IV, and V was decreased in the mubritinib group, the activity of complexes I and III was decreased, and complex V was increased in the cisplatin group. The activity of complex I, III, IV, and V was reduced in the combined group compared to the cisplatin group (Figure 5l–p).

Western blot results showed that the expressions of PI3K, mTOR, p‐Akt, and p‐ERK were downregulated in the monotherapy group (*p* < 0.05), and was further downregulated in the combined group compared with the cisplatin group (*p* < 0.05) (Figure 5r). The expressions of Nrf‐2, HO‐1, and GPX4 were downregulated in the mubritinib group and the expression of GPX4 was downregulated in the cisplatin group. Compared with the cisplatin group, the expressions of Nrf‐2, HO‐1, and GPX4 were down‐regulated in the combined group (Figure 5q).

### Mubritinib promotes the antitumor effect of cisplatin in transplanted tumor

**FIGURE 6:**
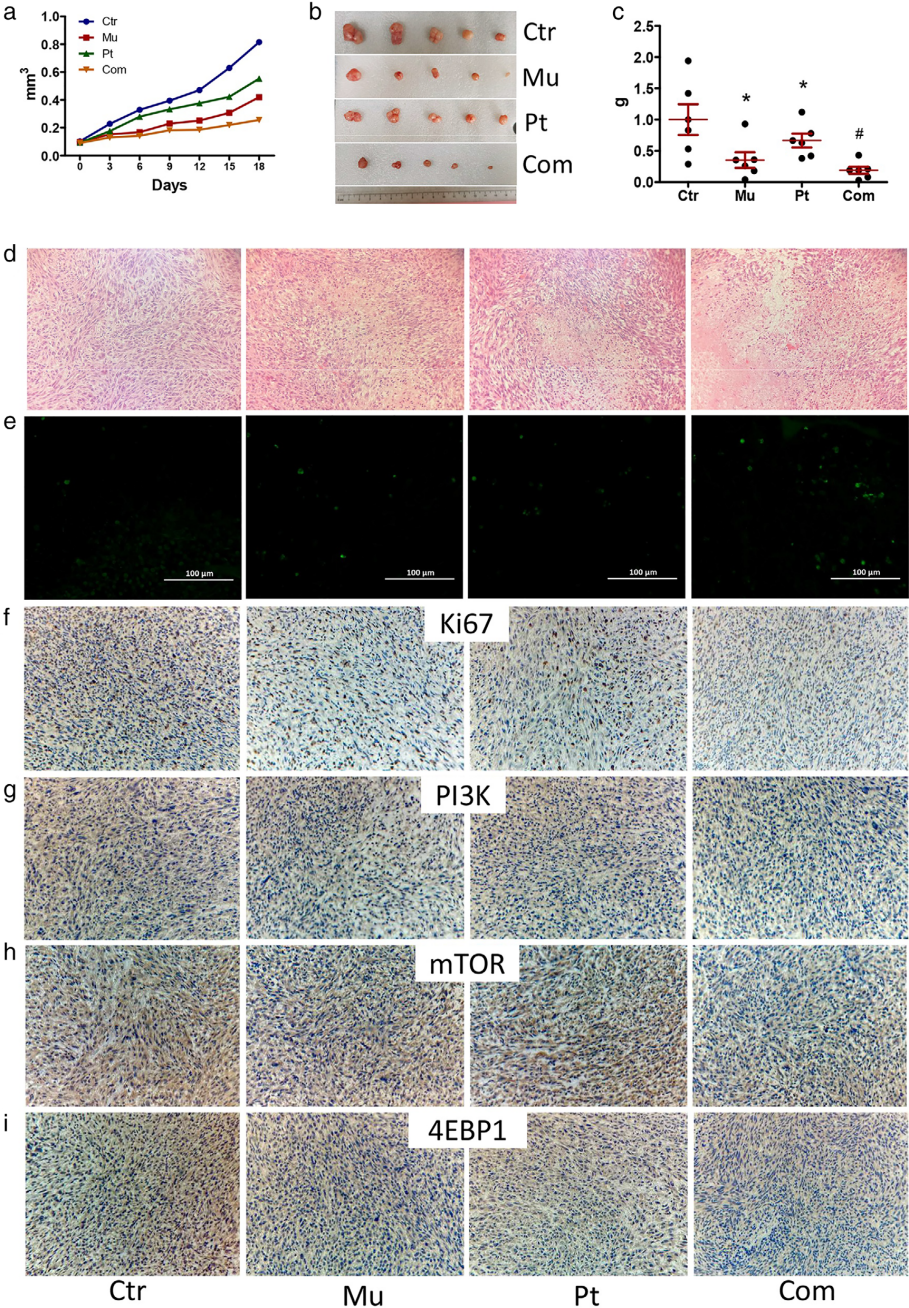
Mubritinib promoted the inhibitory effect of cisplatin in transplanted tumors. (a–c) The growth rate, volume, and weight of transplanted tumors, respectively. (d,e) HE staining and TUNEL fluorescence staining of transplanted tumors, respectively. (f–i) Immunohistochemical staining of Ki67, PI3K, mTOR, and 4EBP1

Compared with the control group, the growth rate of transplanted tumors was inhibited in the mubritinib, cisplatin, and combined groups (*p* < 0.05) (Figure 6a). After 18 days of treatment, the mean volume and weight of grafts were lower in the three treatment groups compared to the control group, especially in the combined group (Figure 6b,c).

**FIGURE 7:**
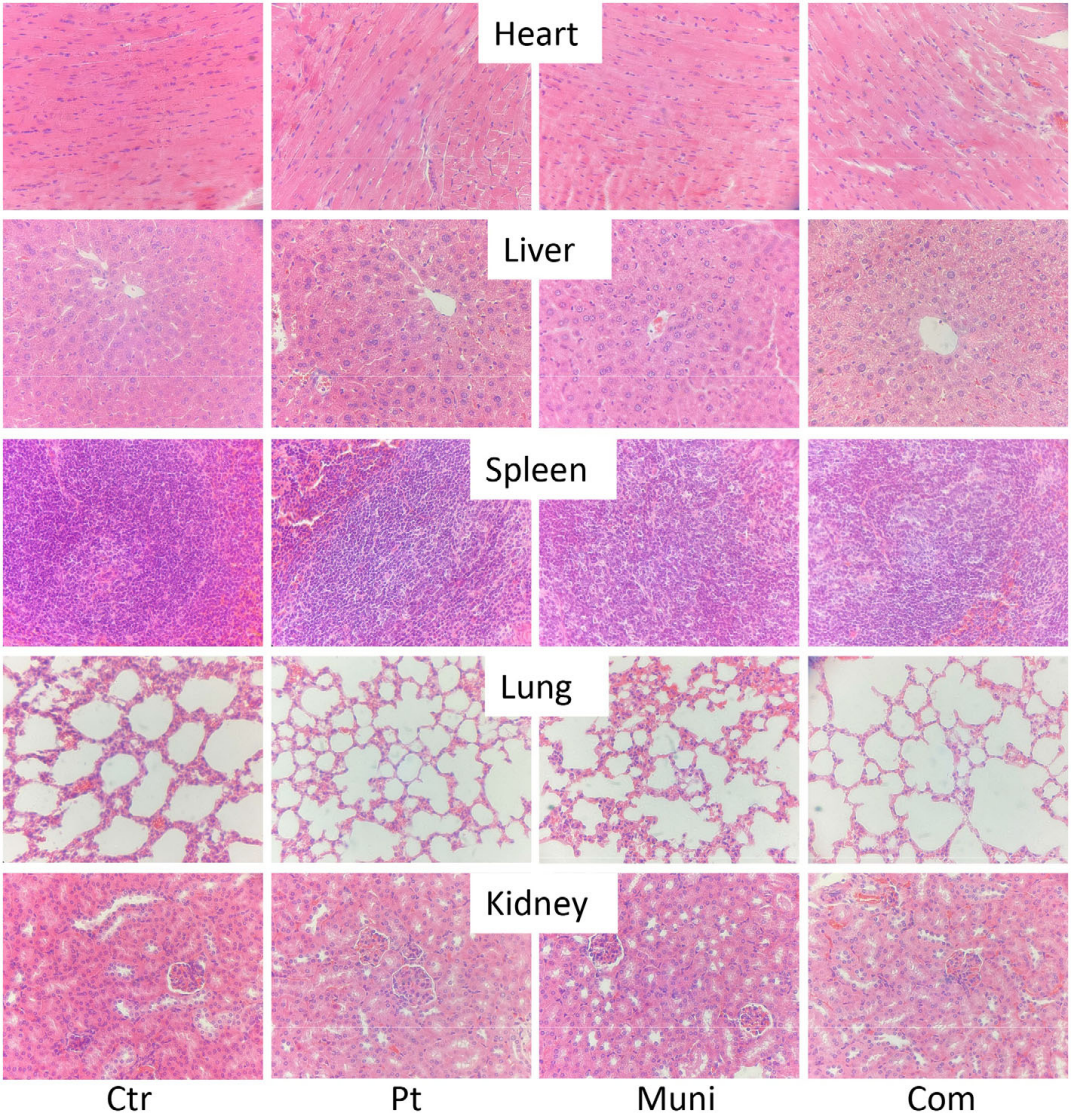
Histology of the heart, liver, spleen, lung, and kidney of mice treated with different treatments

HE staining showed tumor tissue damage, cell reduction, intracellular nuclear pyknosis and nucleolysis, and homogeneous red‐stained necrotic areas were observed in the treatment group with the most obvious damage in the combined group (Figure 6d). In addition, no obvious pathological damage was found in the heart, liver, spleen, lung, and kidney of the four groups (Figure 7).

TUNEL fluorescence staining showed that compared with the control group, the apoptosis rate of cells increased in the treatment groups and further increased in the combined group (Figure 6e). Immunohistochemical staining results showed that the expressions of Ki67, PI3K, mTOR, and 4EBP1 were significantly decreased after cisplatin and mubritinib treatment. Compared with the cisplatin group, the expressions of these proteins were further downregulated in the combined group (Figure 6f–i).

## DISCUSSION

Lung cancer (LC) is one of the most common malignant tumors with an increasing number of cases and deaths worldwide.^8^ At present, the treatment of LC mainly includes surgery, chemotherapy, radiotherapy, targeted therapy, and immunotherapy. Since tumor cells are widely resistant to chemotherapy and targeted therapy, it is necessary to explore new antitumor medicine.

In this study, 42 drugs with inhibitory effects on NCI‐H1975 lung neoplasms cells were screened from the FDA drug library. Mubritinib, which has similar tumor suppressor activity to docetaxel, was selected for the following experiments. Grygielewicz et al.^9^ found that mubritinib can inhibit the SNU‐16R gastric cancer cell line, and Dietel et al.^10^ verified the inhibitory effect of mubritinib on SK‐BR3 breast cancer cells. The results of this study showed that mubritinib had significant antitumor activity on NCI‐H1975 and A549 cells in a dose‐dependent and time‐dependent manner.

To further verify the antitumor effects of mubritinib on LC cells, their apoptosis, proliferation, invasion, and migration abilities were examined. The results showed that mubritinib can promote apoptosis, inhibit proliferation by blocking the cell cycle in the G2 phase, and inhibit the invasion and migration of NCI‐H1975 cells, suggesting that mubritinib may be a candidate drug for lung neoplasms treatment. However, the potential mechanism of action of mubritinib is still unknown. Similarly, Stephenson et al.^11^ found that mubritinib can promote cell apoptosis. Calderon et al.^12^ showed that mubritinib blocked PEL cells in the S and G2 cycles, and selectively increased the G1 phase cell population. Mathavan et al.^13^ reported that mubritinib reduced the migration ability of cells. Inoue et al.^14^ showed that mubritinib inhibited the proliferation and migration of airway epithelial cells.

To further explore the mechanism of action of mubritinib on LC cells, RNA‐seq, also known as transcriptome sequencing technology, was used to reflect the types and amounts of mRNA, small RNA, and noncoding RNA in NCI‐H1975 cells at the transcription level. The analysis showed that the most obvious genetic alteration was thioredoxin interacting protein (TXNIP), which is closely related to the production of cellular ROS and oxidative stress, and further affects the process of apoptosis.^15^ In addition, TXNIP has broad functions in energy metabolism, insulin sensitivity, and tumor suppressor activity in various cancer cells.^15^ KEGG enrichment analysis showed that mubritinib can affect multiple signal pathways, especially the PI3K/Akt pathway, which has been shown to be involved in the malignant transformation, growth, and proliferation of various cancers.^16^

Next, a series of experiments were performed to verify the possible mechanisms mentioned by RNA‐seq. Results indicated that the level of PI3K, p‐Akt, and mTOR in NCI‐H1975 cells were significantly reduced by mubritinib, indicating that mubritinib may inhibit cell proliferation by blocking the PI3K‐Akt–mTOR signaling pathway. The expression of p‐ERK further confirmed this speculation.

Considering that mTOR is a key factor affecting cellular energy metabolism and synthesis,^17^, ^18^ and mitochondria are the main sites for synthesizing ATP and providing energy,^19^ the activities of mitochondrial respiratory chain complex and the mitochondrial membrane potential were investigated. Both the abnormal activity of respiratory chain complexes and the reduction of mitochondrial membrane potential indicated that mubritinib severely impaired the mitochondrial function in NCI‐H1975 cells. Stephenson et al.^11^ found that mubritinib can target mitochondrial respiratory complex I and promote cell apoptosis, while mitochondria mainly control apoptosis by regulating apoptosis‐related proteins, suggesting that the effect of mubritinib on LC cells may be related to mitochondria.

Akt is a serine/threonine kinase that binds the mammalian target of rapamycin (mTOR) complex 2.^20^ Several studies have reported that phosphorylated Akt (p‐Akt) can promote cell proliferation, invasion, metastasis, and angiogenesis, and prevent programmed cell death.^21^ The relationship between this pathway and oxidative stress has also been reported in several studies. For example, Zhuang et al.^17^ reported that resveratrol attenuated the oxidative stress‐induced intestinal barrier damage through the PI3K/Akt‐mediated Nrf2 signaling pathway. In response to ROS damage, the PI3K/Akt signaling pathway is often involved in NRF2‐dependent transcription in a range of cell lines. Zhao et al.^18^ reported that QLQX attenuated the oxidative stress‐induced mitochondrial‐dependent apoptosis in cardiomyocytes through the PI3K‐Akt‐GSK3β signaling pathway. Zhao et al.^19^ found that the oxidative stress‐induced activation of the TGF‐β1‐PI3K‐Akt pathway led to the overproduction of nitric oxide in mesangial cells cultured with high glucose.

In addition, abnormal electron conduction in mitochondrial respiratory chain complexes can increase ROS levels. The detection of intracellular ROS showed that the level of ROS in the mubritinib group was significantly increased, and the expression of Nrf‐2 was upregulated, indicating that NCI‐H1975 cells were subjected to a higher degree of oxidative stress, which in turn activated the antioxidant pathway. The decrease of HO‐1 and GPX‐4 may be due to the increase of ROS and lead to their depletion. Similar to this study, it has been reported that mubritinib can inhibit acute myeloid leukemia by disrupting mitochondrial respiration and inducing oxidative stress and apoptosis.^7^ This new mechanism may provide ideas for new and more effective drug combinations.

Cisplatin, as a key drug in the treatment of LC, delays the survival and life quality of LC patients. However, several disadvantages of cisplatin, including drug resistance, dose‐dependent toxicity, and poor specificity, seriously affect the prognosis of patients.^3^, ^4^ Currently, cisplatin is commonly used in combination with other drugs to improve the effectiveness of tumor therapy.^3^ The results of this study showed that cisplatin combined with mubritinib significantly reduced the proliferation rate and invasion ability of NCI‐H1975 compared with the single‐drug administration group, suggesting that mubritinib and cisplatin have a synergistic antitumor effect.

ROS are byproducts of oxygen consumption and cellular metabolism, formed by partial reduction of molecular oxygen.^22^ Mubritinib combined with cisplatin has previously been reported to significantly increase ROS, decrease mitochondrial membrane potential, and cause oxidative stress and apoptosis in NCI‐H1975 cells. ROS can disrupt mitochondrial membranes and alter their permeability, which has been identified as one of the hallmarks of early apoptosis.^23^ Consistent with these results, Ravi et al.^24^ showed that phytol induces mitochondrial membrane depolarization and activates apoptosis in A549 cells. Mei et al.^25^ showed that targeting mitochondria induces DNA damage‐mediated apoptosis and inhibits the proliferation of HepG2 cells.

Mitochondrial dysfunction can lead to the release of apoptotic factors such as endonuclease G (Endo‐G) and cytochrome C, and therefore activate mitochondria‐mediated apoptosis.^26^ In this study, the activity of mitochondrial respiratory chain complex I, III, IV, and V was significantly reduced after the combined treatment, which is consistent with the results of the study by Guy et al.^7^ In addition, the combined treatment significantly inhibited PI3K/Akt/mTOR signaling pathway, increased the content of ROS, and induced oxidative stress. The PI3K/Akt/mTOR pathway plays an important role in the growth, proliferation, and apoptosis of LC cells.^27^ The activation of the mTOR downstream target, 4EBP1, reduced its affinity with eIF4E and accelerated protein synthesis.^28^

During the experiment, no significant difference in bodyweight and organ morphology was observed among all groups, indicating that the combination of mubritinib and cisplatin was well tolerated.

In conclusion, mubritinib has strong antitumor effects in vivo and in vitro. A better inhibitory effect was observed when it was used in combination with cisplatin by inhibiting the PI3K/Akt/mTOR signaling pathway, increasing the level of cellular ROS, destroying mitochondrial function, and causing cell energy metabolism disorders in NSCLC.

## CONFLICT OF INTEREST

The authors declare that there are no conflicts of interest.

## Supporting information


**Appendix S1** Supporting Information
