# Supplementary material for: Mubritinib enhanced the inhibiting function of cisplatin in lung cancer by interfering with mitochondrial function
Source: Thorac Cancer. 2022 Apr 16;13(10):1513–24. doi: 10.1111/1759-7714.14425 (PMC9108040; doi:10.1111/1759-7714.14425)
Supplement: Supplementary file 1 — Appendix S1 Supporting Information [file TCA-13-1513-s001.docx]

Supplement

Down-regulated genes: TXNIP、FAM111B、SULT1A4、ARRDC4、PPAN-P2RY11、DIO2、PTGES3L-AARSD1、CORO7-PAM16、DEFB4A、TGIF2-C20orf24、ESRP1、LOC105376752、SETDB2、CXCL10、LOC102724788、MAL2、FSBP、TNFAIP8L2-SCNM1、CSNK2A3、FAM101A、MUC5B、TRIM73、LAD1、PCDHA2、EHF、LTA、LOC107987477、U2AF1L5、ITGB6、HPGD、GOLGA6B、EDN2、LOC285556、BEX3、DLX1、GRHL2、ARL14、LOC100996747

Up-regulated genes: GDF15、SULT1A3、ATP5J2-PTCD1、HIST1H4K、MTPN、C4B_2、HRK、C8orf44-SGK3、LOC107987433、LRRC75B、RIMBP3、LOC107984152、C20orf195、ANKRD33、LOC107984862、SYNDIG1L、KIF26A、TMEM110-MUSTN1、PLA2G4B、LOC102724642、SHC2、LTF、GOLGA6C、SLC22A31、RRAGD、LOC107986879、CYP7B1、AATK、LAX1、PGBD3、5-Sep、HHIPL1、USP17L15、TMC6、LPAR5、NGFR、ZNF469、NOVA2、LOC102724197、C7orf57、SNAI3、TBX15、DTX1、ARHGAP30、HSPE1-MOB4、TEDDM1、SMTNL2
